# Extranodal natural killer/T-cell lymphoma, nasal type, involving the skin, misdiagnosed as nasosinusitis and a fungal infection: A case report and literature review

**DOI:** 10.3892/ol.2014.2509

**Published:** 2014-09-08

**Authors:** YAN ZHENG, JINJING JIA, WENSHENG LI, JUAN WANG, QIONG TIAN, ZHENGXIAO LI, JING YANG, XINYU DONG, PING PAN, SHENGXIANG XIAO

**Affiliations:** 1 Department of Dermatology, The Second Affiliated Hospital of Xi’an Jiaotong University, Xi’an, Shaanxi 710004, P.R. China; 2 Department of Pathology, The Third Hospital of Xi’an Jiaotong University, Xi’an, Shaanxi 710068, P.R. China; 3 Department of Dermatology, Chang’an Hospital, Xi’an, Shaanxi 710016, P.R. China

**Keywords:** lymphoma, natural killer/T cell, nasal type, skin perforation

## Abstract

The present study reports a case of extranodal natural killer (NK)/T-cell lymphoma, nasal type, involving the skin. The clinical manifestations, pathological characteristics, treatment and prognosis of the case were analyzed to improve the clinical diagnosis and treatment for this disease. The patient was a 56-year-old male, presenting with dark red nodules and plaques that had been visible on the nose for half a year. Based on the skin lesions and histopathological and immunohistochemical examination results, the patient was diagnosed with extranodal NK/T-cell lymphoma, nasal type. This disease has unique histopathological and immunohistochemical features and a high malignancy. The condition tends to be misdiagnosed and has a poor prognosis, but seldom involves the skin. In the present case, only radiotherapy was performed, with no relapse occurring within 6 months.

## Introduction

Nasal-type extranodal natural killer (NK)/T-cell lymphoma (ENKTCL-N) is an independent-type disease classified as a lymphoid neoplasm by the World Health Organization-European Organization for Research and Treatment of Cancer (WHO-EORTC) 2005 standard (1) (the 2008 standard being the final standard). ENKTCL-N most commonly involves the nasal cavities, paranasal sinuses and nasopharynx (2). This disease is characterized by clinical rarity, rapid progression and a high mortality rate, but its uncharacteristic early clinical manifestations and infrequent skin involvement tend to result in missed diagnoses and misdiagnosis (3). The present study reports a case of ENKTCL-N involving the skin that was diagnosed at the Department of Dermatology, the Second Affiliated Hospital of Xi’an Jiaotong University (Xi’an, China), combined with a literature review. Written informed consent was obtained from the patient for the publication of the present study and any associated images.

## Case report

### Patient and case history

A 56-year-old male presented to the Department of Dermatology, the Second Affiliated Hospital of Xi’an Jiaotong University with a nodule on the left side of the nose, which had been progressively enlarging for one year. One year previously, a pimple approximately the size of a grain of rice had appeared in the patient’s left nasal cavity without evident inducement. The pimple caused no itching or pain, and the corresponding external skin on the nose exhibited no noticeable change, so the patient paid no attention to the pimple. Subsequently, the nodule gradually grew. Six months prior to the current presentation, the patient noticed that the nodule was locally red, swollen and erosive, and the patient felt mild nasal congestion and had difficulty breathing. The patient was diagnosed with chronic nasosinusitis at the Ear, Nose and Throat Department of a local hospital. Subsequent to receiving cephalosporin antibiotics as an anti-inflammatory treatment, the redness and swelling subsided, but the nodule remained. Afterwards, the nodule continued to enlarge progressively and grew internally and externally, accompanied by hyperemia and swelling of the corresponding skin outside the nose. Three months prior to the current presentation, upon agitation by the patient, the nodule ulcerated and oozed blood, with locally damaged skin, deteriorated nasal obstruction and a large amount of purulent nasal discharge. However, the patient did not experience itching and pain or systematic symptoms, such as fever and emaciation. The patient returned to the local hospital. This time, the patient was diagnosed with a nasal fungal infection and received oral itraconazole (200 mg, twice a day) for two weeks. Although the local swelling was somewhat alleviated and the bleeding stopped, the nodule did not significantly decrease in size. The subsequent external use of topical amphotericin B (twice a day, for one week) slightly mitigated the condition and the patient arrived at our hospital for a diagnostic examination. Since the onset of the symptoms, the patient had experienced no changes in mood, diet, sleep, urination or defecation, and no marked changes in body weight. Prior to the onset of the condition, the patient had no history of local trauma. The patient was normally healthy and had smoked for 38 years (20 cigarettes/day). The patient’s mother had previously been diagnosed with colorectal cancer and the patient’s son had been diagnosed with a pituitary tumor. The remaining family members were all healthy and no similar diseases were reported.

### Physical examination

**Figure 1:**
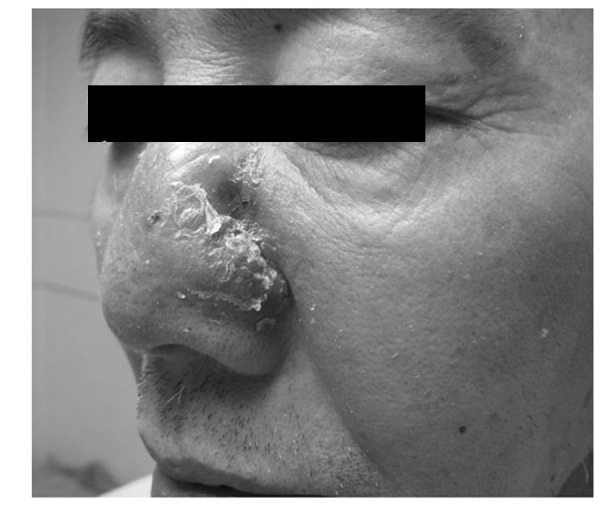
Image of the nodule upon initial presentation.

**Figure 2:**
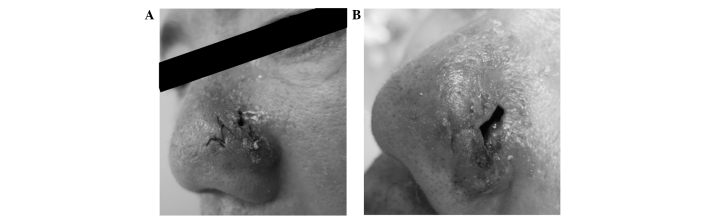
(A) Image of the nodule captured one week after the biopsy procedure. (B) One week after the biopsy procedure, following the removal of the stitches.

The patient possessed stable vital signs, the systemic superficial lymph nodes, liver and spleen were not involved in the disease, and the systematic examination revealed no evident abnormalities. A dermatological examination revealed that the nose was asymmetric and that the right side was normal. Wet red plaques (~4×5 cm) were present on the left side of the nose, with scales on the surface that were hard and painful when touched, without crepitus or fluctuation. Irregular defects, ~1 cm long, reached the nasal cavity, with a small number of purulent secretions on the surface (Figs. 1 and 2). The left nasal cavity exhibited numerous sticky black scabs that were difficult to remove, tended to bleed and generated malodorous secretions, which the patient could not detect, and the structure of the nasal cavity was indiscernible. The right nasal cavity had a small number of scabs and secretions and exhibited mucosal atrophy. The nasopharyngeal mucosae were smooth and retained a large number of mucus secretions. The left maxillary sinus area became swollen and painful when pressed.

### Auxiliary examination

**Figure 3:**
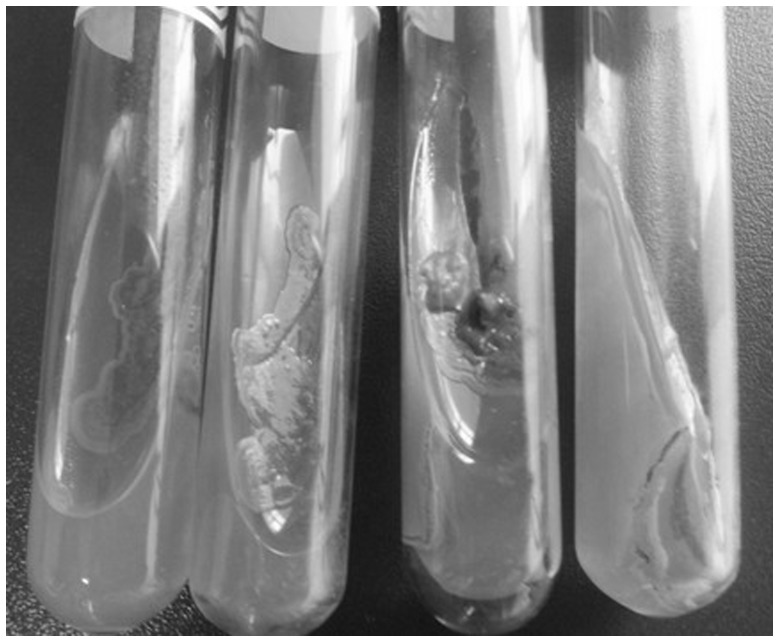
Fungal culture with negative results.

**Figure 4:**
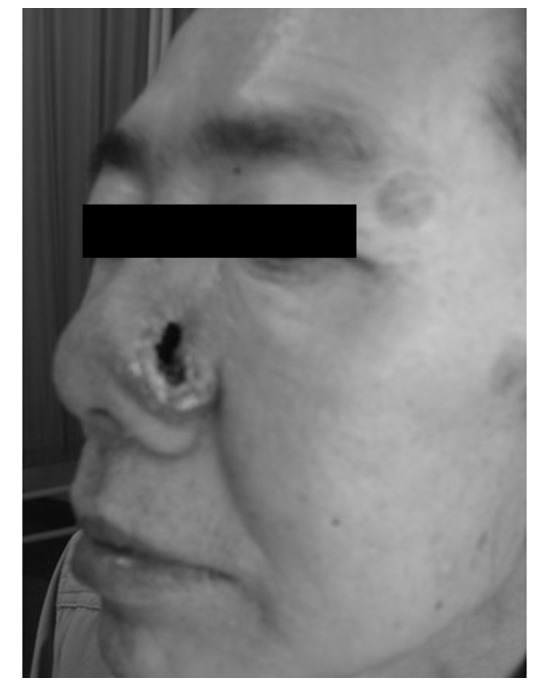
Two weeks after the biopsy procedure. The damaged skin around the lesion site demonstrated improvement, but the size of the ulcer had increased.

Fungal cultures of the nasal secretions and the lesion tissue blocks each revealed negative results (Fig. 3). Paranasal sinus computed tomography (CT) plain scanning revealed that the soft tissue of the lateral wall of the left nasal cavity was thickened, with a discontinuous shape, and that its inner density was moderate. The left middle nasal meatus and a section of the right nasal cavity were involved. The subcutaneous soft tissues of the left zygomatic arch was swollen and the mucosae of the left ethmoid sinus and the maxillary sinus were thickened. Two weeks later, the lesion had improved, however the size of the ulcer had increased (Fig. 4).

### Histopathological changes

**Figure 5:**
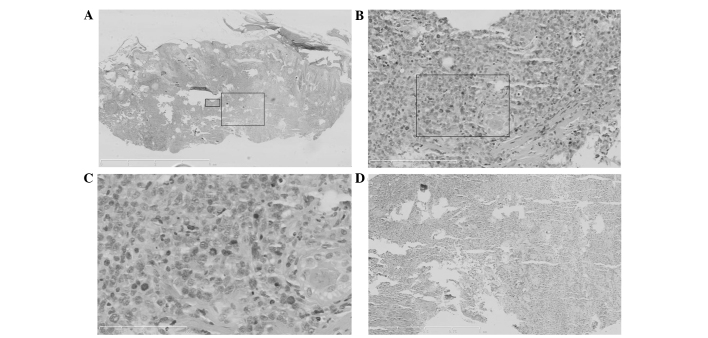
Biopsy samples from the left wing of the nose. (A) Dermis exhibiting epidermal necrosis and erosion, hyperkeratosis of the skin, side dyskeratosis, crusting and epidermal angle extension. Dermal shallow central vascular dilatation and extravasation of erythrocytes is also present. In addition, infiltration of the lymphocytes, plasma cells and neutrophils into the dermis, with profuse necrosis, may be observed (bar = 4 mm). The extent of image 5(B) is indicated by a small black boxand the extent of image 5(D) is indicated by a larger black box. (B) The specific lymphocytes in the lower dermis and subcutaneous sections of the biopsy sample (bar = 200 μm). The majority of large cells, which positively stained for CD56 were NK cells and the majority of small cells were T-cells. There are more medium and large cells with thick nuclear membranes. The chromatin within the cells is fine and the plasmosomes are not evident. Mitotic phases are more common and are attached to vessels. The extent of image 5(C) is indicated by a large black box. (C) Image (bar = 100 μm) (B) at a higher magnification. (D) Large areas of necrosis and infiltration of neutrophils (bar = 1 mm). Hematoxylin and eosin staining.

A section of the epidermis was necrotic and erosive. The side with the lesion exhibited hyperkeratosis, parakeratosis and epidermal cutin extension. Numerous abnormal lymphoid and plasma cells, and extensive neutrophile granulocyte infiltration could be observed at the depth of the dermis and the subcutaneous adipose tissue, together with large areas of necrosis and commonly observed broken nuclei. Diffuse infiltration was present. The abnormal lymphoid and plasma cells were of various sizes, but mainly medium and large. The cells were characterized by a thick nuclear membrane, fine and smooth chromatin, inconspicuous nucleoli, commonly observed nuclear fission and an angiotropic phenomenon (Fig. 5).

### Immunohistochemistry

**Figure 6:**
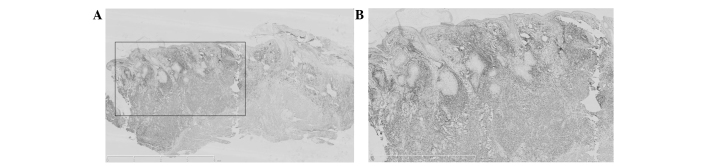
(A) Biopsy sample positive for cytoplasmic cluster of differentiation (CD)3ɛ expression. The extent of image (B) is indicated by a black box. (B) Magnification of the black box in image (A). CD3ɛ-cytoplasmic-positive results. (A) Bar = 4 mm. (B) Bar = 2 mm.

**Figure 7:**
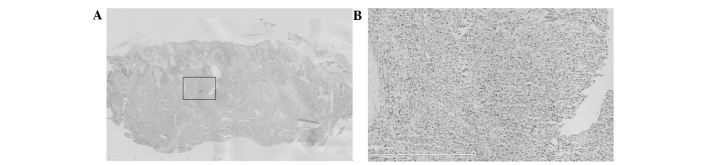
(A) Biopsy sample positive for Epstein-Barr virus-encoded small RNA (EBER) expression (bar = 4 mm). The extent of image (B) is indicated by a black box. (B) Magnification of the black box in image (A) (bar = 500 μm). EBER-positive results.

**Figure 8:**
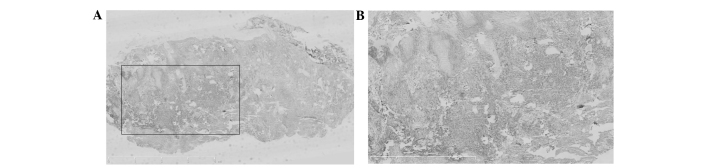
(A) Biopsy sample positive for cluster of differentiation (CD)56 expression (bar = 4 mm). The extent of image (B) is indicated by a black box. (B) Magnification of the black box in image (A) (bar = 2 mm). CD56-positive results.

**Figure 9:**
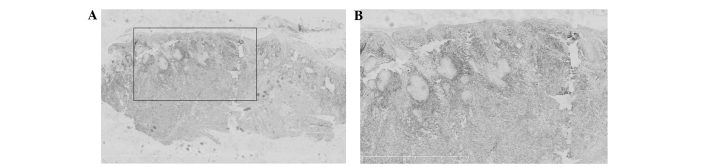
(A) Biopsy sample positive for granzyme B expression (bar = 4 mm). The extent of image (B) is indicated by a black box. (B) Magnification of the black box in image (A) (bar = 2 mm). Granzyme B-positive results.

**Figure 10:**
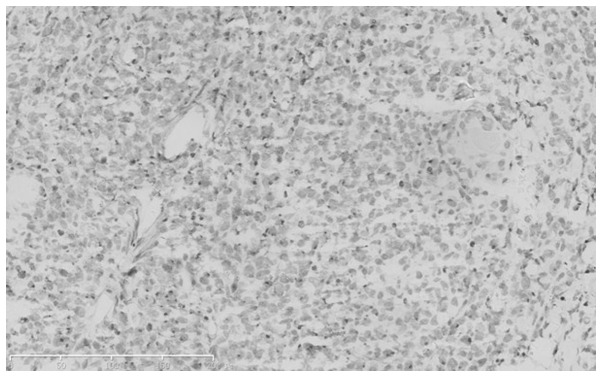
Biopsy sample positive for TIA1 expression. Bar = 200 μm.

**Figure 11:**
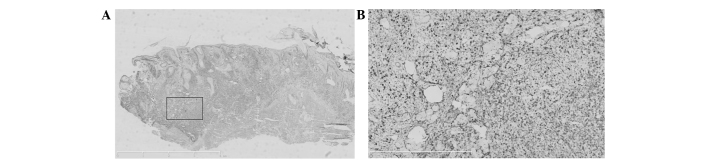
(A) Biopsy sample with a Ki67 proliferation index of 60–70% (bar = 4 mm). The extent of image B is indicated by a black box. (B) Magnification of the black box in image (A) (bar = 2 mm). Ki67 index of 60–70%.

**Figure 12:**
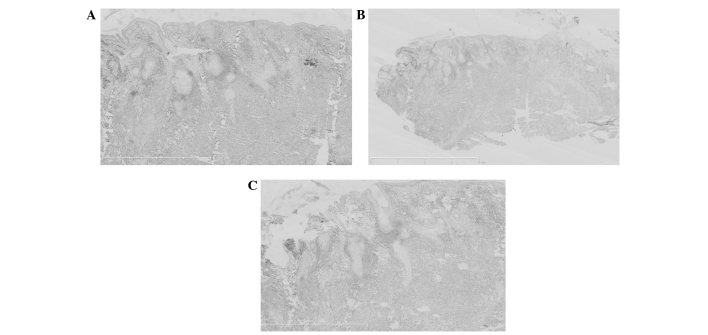
Biopsy samples negative for (A) cluster of differentiation (CD)5 (bar = 2 mm), (B) CD20 (bar = 4 mm) and (C) CD2 expression (bar = 1 mm).

The tumor cells revealed a positive immunoreaction for cluster of differentiation (CD)3ɛ, CD56, Epstein-Barr virus (EBV)-encoded small RNAs, granzyme B and TIA1, with a Ki67 of 60–70%. The immunoreaction was negative for CD5 and CD20 (Figs. 6–12).

### Diagnosis

The patient was diagnosed with ENKTCL-N (super-cavity stage IE) (4,5).

### Treatment

**Figure 13:**
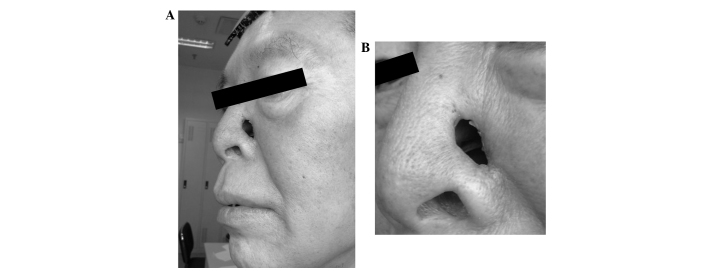
(A) Lesion site one month after treatment. (B) Magnified version of image (A) showing the oval defect and healed scar.

Radiotherapy treatment was adopted following the confirmation of the diagnosis. The radiotherapy program was carried out using a Siemens ONCOR linear accelerator (Siemens, Munich, Germany) for image-guided radiotherapy (IGRT). The gross tumor volume (GTV) consisted of the left nasal cavity lesion visible on CT. The clinical target volume (CTV) consisted of the GTV and the right nasal cavity, the wings of the nose, the left maxillary sinus and the ethmoid sinus. The planning target volume (PTV) consisted of the CTV and the surrounding 0.5-cm area. Three-field isocentric irradiation, with the PTV as the target, was conducted at 2 Gy/fraction, 5 times/week, with a total of 60 Gy/30 fractions. During the treatment, the patient was required to look straight ahead, with an open mouth and a tongue depressor in place, and with fillers in the nose to improve the GTV skin dose. During the radiotherapy, eye cleaning and local washing of the GTV were intensified. In addition, the patient topically applied mupirocin (twice a day, for two weeks) to diminish inflammation, received oral vitamin C (200 mg, twice a day) and vitamin B (5 mg, twice a day) to alleviate the mucosa reaction and used Traditional Chinese Medicine to strengthen the body’s resistance and to maintain vitality. Following the radiotherapy, the patient experienced normal health. The skin of the left wing of the nose was normal, except for a 1×0.5-cm regular defect reaching the nasal cavity, from which the patient felt no pain when pressure was applied. The mucosae in each of the nasal cavities were smooth. An oval defect with an area of ~1×2 cm^2^ could be observed beneath the left wing of the nose and the scar had healed two weeks following treatment (Fig. 13). Following the treatment, the patient achieved complete remission (CR), with no relapse during the six months of clinical follow-up.

## Discussion

According to the 2008 WHO-EORTC classification of lymphoid neoplasms (1), ENKTCL-N belongs to the category of mature T-cell and NK cell lymphomas.

Since the vast majority of tumor cells express a NK cell phenotype and only a small number of cells express a cytotoxic T-cell phenotype, the majority of ENKTCL-Ns are considered to be derived from mature NK cells, and only a fraction of them derive from NK T cells (6). Therefore, the condition has been termed NKTCL.

ENKTCL-N occurs predominantly in middle-aged males, with a male to female ratio of 3:1 and a median age of 44 years. The disease is more common in Asia, particularly in China and Japan, and South America, where it accounts for 7–10% of all non-Hodgkin’s lymphomas. ENKTCL-N is less common in Western countries, accounting for 1% of all non-Hodgkin’s lymphomas (7), suggesting a geographic or ethnic susceptibility to the disease.

EBV is a human DNAγ herpes virus. Latent membrane protein-1 (LMP-1) is an identified tumor protein encoded by EBV, which plays a key role in regulating the process of the malignant transformation of cells (8). Present studies suggest that patients with NKTCL-Ns have an EBV infection rate of >90%, while the infection rate of patients with non-nasal NKTCLs exhibits a downward trend (9,10). Certain studies have hypothesized that EBV infection is site-dependent, but there are also findings that are not consistent with this viewpoint (11–13). NKTCL predominantly occurs in the nasal cavities, and the upper respiratory tract is the infection channel of EBV, so the EBV detection rate is relatively high (14). Therefore, it is necessary to further investigate and discuss whether EBV infection is site-dependent or if it has a high affinity for this type of lymphoma.

Pesticides and chemical solvents can be observed as possible factors in the pathogenesis of NKTCL, suggesting that lifestyle and environmental factors are closely associated with NKTCL (12).

Currently, the focus of the investigation into the abnormal aspects of the NKTCL-N chromosome includes deletion on the short arm of chromosome 6, deletion on the long arm of chromosome 1, extension of the short arm of chromosome 2 and occasionally includes isochromosome 7q (15,16). In addition, the p53 gene mutation rate in NKTCL-N is 24–48%, higher than those of the other lymphomas, but there is no correlation between EBV and p53 mutation (17). Fas is a cell surface receptor that participates in cell death signaling by binding the Fas ligand, and mutation of the Fas gene often results in the accumulation of lymphocytes, thus causing tumors (18). A previous study reported that half of a group of 14 NKTCL-N cases possessed a mutation in the Fas gene, suggesting that Fas gene mutation is the pathological basis of NKTCL and that its mechanism of action involves the inhibition of the apoptosis of lymphocytes (19). Other genetic abnormalities include abnormal expression of c-kit, β-catenin and other proto- and anti-oncogenes, B-cell lymphoma (Bcl-2) protein overexpression, low or negative expression of the p73 gene, a novel member of the p53 gene family, and upregulated survivin gene expression, which is negatively correlated with the apoptosis index. The association between these gene mutations and EBV remains unclear (20).

Ki-67 is highly expressed in ENKTCL-N, with a much higher expression level compared with the level in follicular lymphoma (P<0.001). The Ki-67-positive rate of tumor cells in the majority of ENKTCL-N patients is >60% (19). Certain studies have identified that, among ENKTCL-N patients, the higher the Ki-67 proliferation index, the worse the prognosis (22). Other relevant abnormal expression proteins have also been reported, including nuclear factor κB, chemokine ligand 9, G-protein signaling regulator 2, Bcl-2, induced myeloid leukemia cell differentiation protein, platelet-derived growth factor receptor and ubiquitin activating enzyme E1 (23–27).

The clinical features of ENKTCL-N occurring in the nasal cavities, include the clinical symptoms of nasal obstruction, rhinorrhea with blood or epistaxis, tinnitus, hoarseness, pharyngalgia and discomfort whilst swallowing. The physical signs of ENKTCL-N include redness and swelling, ulceration and even penetration of the local tissues, or tumefied lumps that are often malodorous (28). Progressive development may involve the eye sockets, cheeks and frontal bone. The most prominent facial feature is damage to the midline areas, including nasal septum perforation, hard palate perforation and nasal bridge perforation (3). This disease has rapid clinical progression and extremely high invasiveness, and the subsequent diffusion may involve the skin, gastrointestinal tract, testes, central nervous system, spleen and other organs and tissues, but rarely the lymph nodes (29). The bone marrow and circulatory system may also be involved; in this situation, the disease overlaps with aggressive NK-cell leukemia, which is actually the end-stage manifestation of the disease, and the two have the same immunophenotype and genotype (28). In the present study, ENKTCL-N involved the skin, leading to a skin ulcer and perforation, and the patient only received radiotherapy, with no relapse occurring within seven months.

ENKTCL-N exhibits numerous histological characteristics. The tumor cell infiltration position is deep, mainly in the dermal and subcutaneous adipose tissues, and the tumor cells exhibit microvascular invasion and damage, with coagulative necrosis and apoptosis being common. The tumor cells vary in size, including small-, medium- and large-sized cells, with the majority of cases being dominated by medium-sized cells together with varying numbers of small- and large-sized cells (30). The cells have irregular nuclei and nuclear fission is commonly observed. There is often infiltration of the inflammatory cells, including small lymphocytes, neutrophils, histiocytes and eosinophils mixed in neoplastic lymphocytes, so the disease tends to be misdiagnosed as inflammation (2,31). Mraz-Gernhard *et al* (32) hypothesized that the angiocentric invasion and pro-epidermal phenomenon was not a feature of NK/T and that this was the most useful histological characteristic in distinguishing cutaneous NKTCL from other cutaneous lymphomas (32).

The immunophenotype of ENKTCL may be primarily characterized by a lack of CD3 and CD20 expression, positive CD3ɛ, CD2 and CD56 expression, positive TIA-1, granzyme B and perforin expression and positive EBER expression.

The main basis for the diagnosis of ENKTCL-N is the clinical pathogenic locations combined with the histopathological and immunohistochemical examination results. However, the uncharacteristic early manifestation of the disease tends to result in misdiagnosis.

There are several reasons for misdiagnosis. The first is that the early manifestation has no special features and is dominated by nasal congestion, rhinorrhea, rhinorrhea with blood and other chronic nasosinusitis symptoms. Additionally, due to the small scope of early lesions and the indetectable lesion locations, doctors and patients may pay no attention to them, and only conduct simple treatment or even abandon treatment. Another reason is that during pathological examination, the negative detection rate is relatively high. The disease is angiotropic and tends to cause vascular occlusion and consequent tissue necrosis within the scope of the blood supply; improper sampling may lead to a large number of necrotic tissues under the microscope. In addition, tumor cells vary greatly and are not typical in size, including large-, medium- and small-sized cells. In the case of concurrent infection and over-small or over-shallow specimens, doctors may only see inflammatory cells and often diagnose the disease as necrotic tissue with inflammatory infiltration (33). A final reason is that lymph node metastasis and distant spread is generally late-stage, and the patients are in a generally good condition, so the disease-symptom separation tends to render patients and doctors careless (33,34).

ENKTCL-N must be differentiated from several diseases, including inflammatory lesions, which are characterized by extensive inflammatory cell infiltration, with ulceration in certain lesions, but without abnormal lymphocyte proliferation or vascular invasion and damage. Hybridization *in situ* reveals that the cells are negative for EBER expression. Additionally deep mycoses must be considered. It is difficult to distinguish clinical mycoses and deep mycoses. The upper section of the pathological dermis consists of mixed cell infiltration, but the middle and lower sections of the dermis have subcutaneous abnormal lymphocytes, and immunohistochemistry and fungal culture aids differentiation. There is mixed inflammatory cell infiltration, with no abnormal cells, and the fungal culture is positive (35).

ENKTCL-N must also be differentiated from Wegener’s granulomatosis. The main feature of Wegener’s granulomatosis is necrotic granulomatous inflammation and true vasculitis. Inflammatory cells invade the entire vascular wall, causing elastic fiber damage, vascular occlusion or fibrinoid necrosis, with no abnormal tumor cells. Elastic fiber staining aids differentiation. Furthermore, cutaneous γδ T-cell lymphoma requires differentiation. Immunohistochemical investigation reveals the cells to be CD2-, CD3- and CD7-positive, T-cell toxic granule-positive, and generally CD4-, CD8-, and EBV-negative (36). In addition, ENKTCL-N must be distinguished from aggressive pro-epidermal CD8-positive cytotoxic T-cell lymphoma. The two diseases are primary cutaneous peripheral T-cell lymphomas and are rare. Pro-epidermal lymphoma is negative for EBV and positive for CD8 expression (37). ENKTCL-N and blastic plasmacytoid dendritic cell neoplasms must also be differentiated. Blastic plasmacytoid dendritic cell neoplasms mainly involve the skin, with leukemoid dissemination. The neoplasm tumor cells exhibit positive CD4, CD56 and CD123 expression, but are negative for CD3, TIA-1 and EBV expression. Adult T-cell leukemia and lymphoma are dominated by peripheral T-cell tumors composed of highly pleomorphic lymphoid neoplasms. The skin is the most common site of extranodal involvement. Tumor cells express CD25 and forkhead box P3 at the same time. Tumor involvement is common in the peripheral blood, with immunophenotypes primarily characterized by negative CD56 and EBER expression (38).

Patients with early-stage (Ann Arbor stage I–II) NKTCL-N are more sensitive to radiotherapy compared with stage III–IV patients, and single-line radiotherapy has a good effect and is the main method of treatment (39,40). The radiotherapy consists of a daily dose of 1.8–2.0/2.5 Gy (5 times/week), a total irradiation dose range of 20–70 Gy and a median dose of 45 Gy. The irradiation fields include the primary site and a sufficient edge region, as well as the nose, paranasal sinuses, nasopharynx and pharyngeal lymph ring (Waldeyer’s ring) (41,42). If the cervical lymph node is involved, the irradiation range can be extended to the neck, and where the nasopharynx or pharyngeal lymph ring is the primary site, patients should receive conventional irradiation, regardless of whether the cervical lymph nodes are involved (43). A previous study reported that, soon after radiotherapy, patients achieved a CR, with a reported CR rate of 70% and an effective rate of 80% (20). Compared with low-dose radiotherapy, large-dose irradiation (≥50Gy) has a better effect, with five-year local control rates of 100 and 67%, respectively; this difference is statistically significant (P=0.013) (40).

Although radiotherapy has clear short-term effects, it is not applicable to disseminated or relapsing cases, and a large number of studies have revealed an extremely high long-term relapse rate for single-line radiotherapy (range, 17–77%, with 50% as the most commonly reported value). This indicates that single-line radiotherapy may be insufficient for even stage I and II patients, and current attention should be focused on whether the combination of radiotherapy and chemotherapy can aid the improvement of the curative effect (40).

For patients with early-stage (stage I and II) ENKTCL-N, single-line radiotherapy retains high local relapse and distant tumor dissemination rates (40,44). Currently, these patients are mainly treated by combining radiotherapy and chemotherapy, including radiotherapy followed by chemotherapy, chemotherapy followed by radiotherapy, the sandwich method (2–4 courses of chemotherapy, followed by radiotherapy and then chemotherapy for the remaining courses of treatment) and concurrent radiotherapy and chemotherapy (45). Theoretically, the combination of radiotherapy and chemotherapy not only administers early-stage radiotherapy to local lesions, but it also targets distant potential metastatic lesions. You *et al* (46) investigated the treatment program for NKTCL-N in 46 patients. The results revealed that chemoradiotherapy is more effective compared with radiotherapy and chemotherapy. Yang Yong *et al* (47) reviewed and analyzed 18 cases of patients with early-stage ENKTCL-N. The 18 cases consisted of three cases of induction chemotherapy with concurrent chemoradiotherapy and adjuvant chemotherapy, 13 cases of induction chemotherapy with concurrent chemoradiotherapy, one case of concurrent chemoradiotherapy with adjuvant chemotherapy and one case of concurrent chemoradiotherapy. The study used sequential chemoradiotherapy as the control. The results revealed five-year overall survival rates of 80.8 and 54.3% in the concurrent chemoradiation therapy and control groups, respectively, suggesting that concurrent chemoradiotherapy may improve the long-term survival of early-stage patients (46).

For patients with early-stage (stage III and IV) ENKTCL-N, the treatment programs are dominated by systemic chemotherapy, and radiotherapy is only used as an auxiliary control for local lesions. However, for advanced-stage tumors, numerous types of treatments are of poor curative effect, and stronger or novel effective treatment programs require consideration (48).

The treatment of NKTCL by hematopoietic stem cell transplantation (HSCT) remains in the exploratory stage. In recent years, the method of high-dose chemotherapy followed by auto-HSCT has achieved good results. Pre-HSCT high-dose chemotherapy can mobilize a sufficient number of hematopoietic stem cells, kill *in vivo* tumors, avoid the reinfusion of stem cells contaminated by tumors and realize *in vivo* ‘purification’ (49). Niu *et al* (50) treated a female patient with invasive ENKTCL-N by auto-HSCT. During the 67 months of follow-up, the patient remained in CR.

Currently, the investigation into NK/T-cell lymphoma targeted therapy has just begun; there has been no targeted drug entering clinical trials, and bortezomib, a type of proteasome inhibitor, has demonstrated curative effects in certain preliminary studies (51). However, Kim *et al* (52) reported the treatment of 46 T-cell lymphoma patients using bortezomib combined with the cyclophosphamide, doxorubicin, vincristine and prednisolone chemotherapy program, including 10 cases of ENKTCL-N, eight cases of angioimmunoblastic T-cell lymphoma, six cases of anaplastic lymphoma kinase-negative anaplastic large cell lymphoma, five cases of cutaneous T-cell lymphoma, one case of hepatorenal T-cell lymphoma and 16 cases of non-independently classified peripheral T-cell lymphoma. The results revealed that a total of 30 patients (CR rate, 65%) achieved a CR following treatment, but of the 10 cases of ENKTCL-N patients, only three cases (CR rate, 30%) achieved a CR following treatment. This rate was far lower than that for other subtypes of T-cell lymphoma (CR rate, 73%). The aforementioned results are different from *in vitro* studies (44), which indicate that bortezomib has an anti-NK lymphoma cell effect, suggesting that the targeted therapy for ENKTCL-N requires further study (52).

Nasal-type NKTCL is a highly aggressive tumor with a poor prognosis, a median survival time of <12 months and a previous five-year survival rate of 30–40%. However, this five-year survival rate has increased to 71% (28) in recent years with the application of intensive treatment methods. Studies have demonstrated that the prognosis of patients is associated with numerous factors, including bone marrow infiltration, tumor invasion, existence of group B symptoms (fever >38°C for >3 days, night sweats and weight loss of >10% in six months), EBV DNA level of circulating blood (53), granzyme B, CD94, Ki-67 expression and hemoglobin concentration prior to treatment (54). Clinically, the most commonly used risk assessment system with a proven prognosis value is the international prognostic index (55) and certain Korean scholars have introduced the Korean prognostic index, which includes group B symptoms, Ann Arbor staging, lactate dehydrogenase levels and the involvement of 1–3 lymph nodes, but without distant transfer (56). All the aforementioned methods provide guidance for the prognosis assessment of ENKTCL-N patients (55).

The patient in the present study was a middle-aged male with a long history of heavy smoking. Starting with a nodule on the nose, the patient only experienced nasal congestion, shortness of breath and other non-specific symptoms in the early stages, so paid no attention to the condition for a long time. Later, since the patient had the habit of picking at his nose, the progressive development of the disease and frequent agitation of the nodule led to local redness, swelling and erosion, and then even skin ulceration and bleeding. However, there was no itching, pain or lymphoma group B symptoms, which include a fever >38°C for >3 days, night sweats and weight loss of >10% in six months (4,5). Since the patient was in a good general condition, the symptoms were non-specific, a local secondary infection had occurred and the antibiotics and antifungal agents had exhibited a limited effect, the patient was misdiagnosed with nasosinusitis and nasal fungal infection several times. However, the tumor maintained its progressive development instead of being effectively controlled. Combining the results of histopathological and immunohistochemical examinations, the Department of Dermatology, the Second Affiliated Hospital of Xi’an Jiatong University eventually diagnosed the patient with ENKTCL-N. In addition to the primary site, the lesions also invaded the local skin, and left maxillary, left ethmoid and right ethmoid sinuses, without lymph node or distant metastasis. The nodule was an early-stage lymphoma (super-cavity IE period) and the patient was sensitive to radiotherapy, so high-dose (60 Gy) IGRT was adopted. In order to prevent long-term relapse and achieve better long-term survival, additional systemic chemotherapy was suggested, but the patient refused due to the possible side-effects. The radiotherapy achieved a good response and the patient achieved a CR following the treatment. During the six months of clinical follow-up, no relapse occurred. It was recommended that the patient regularly undergo follow-up examinations, including blood routine examinations, paranasal sinus CT, chest CT, abdominal ultrasonography and pelvic CT, in order to detect a local relapse or distant metastasis early and then undergo chemotherapy.
